# Parents Without Partners: *Drosophila* as a Model for Understanding the Mechanisms and Evolution of Parthenogenesis

**DOI:** 10.1534/g3.112.005421

**Published:** 2013-04-01

**Authors:** Therese Ann Markow

**Affiliations:** Section of Cell and Developmental Biology, Division of Biological Sciences, University of California at San Diego, California 92093-0116, and Laboratorio Nacional de Genomica de la Biodiversidad, CINVESTAV, Irapuato, Guanajuato CP 36821, Mexico

## Abstract

Of 40 *Drosophila* species screened to date, a majority have shown some ability to at least initiate parthenogenetic development. In one case, *Drosophila mangebeirai*, natural populations are entirely female, making it the only obligate parthenogenetic species of *Drosophila*. Only a few of the species that exhibit the ability to undergo early embryonic development of unfertilized eggs successfully respond to selection for parthenogenetic production of adult flies. Laboratory strains of parthenogenetic *Drosophila mercatorum* have been created by artificial selection on multiple occasions, but the proportion of eggs undergoing development to adulthood has never exceeded 8%. Selection produces gains in the number of unfertilized eggs undergoing early development, but the majority arrest at the embryonic or first larval instar stages. Four components to successful parthenogenesis include (1) a female’s propensity to lay unfertilized eggs, (2) the ability of the eggs to restore diploidy, (3) the ability of the parthenogenetically produced diploid embryo to complete larval development and pupation, and (4) the existence of genetic variability within and among *Drosophila* species in the frequency of parthenogenesis suggests the existence of multiple steps in its evolution and offers a way to explore the genetics of this unusual reproductive strategy.

Parthenogenesis occurs in a wide range of animal taxa and is produced by a variety of different underlying mechanisms. Parthenogenesis was not the ancestral state in the taxa where it occurs; at some point in their evolutionary histories, genetic variants must have arisen that (1) allowed impaternate development of embryos, and (2) were favored by existing ecological or demographic factors. Accurate reconstruction of past evolutionary events is difficult, and determining the origin of parthenogenetic taxa is no exception. However, species in the very earliest stages of becoming parthenogenetic, or facultatively parthenogenetic, can provide insights into the mechanisms and selective forces that have permitted, and perhaps even driven, the evolution of extant parthenogenetic species.

Parthenogenesis in *Drosophila* first caught the attention of Stalker (Stalker 1951, 1952) during studies of reproductive isolation among species in the cardini species group. He observed that virgin females of both *Drosophila polymorpha* and *Drosophila parthenogenetica* produced, at low levels, all female progeny, which went on to produce, in the absence of males, all female offspring. Parthenogenesis since has been screened for in 40 *Drosophila* species (Table 1). The table is a compendium of studies and the species listed were surveyed with different approaches. In some cases wild-caught females or their F1 virgin daughters were tested whereas other investigations examined virgin females from laboratory stocks of various genotypes. Sample sizes vary enormously, from more than 10 million eggs in *Drosophila robusta* to only 2600 in *Drosophila macrospina*. Given that parthenogenetic development is rarer in some species, the current picture therefore is likely an underestimate. With one exception, *Drosophila mangabeirai*, parthenogenetic development is facultative in all species where it is observed. Furthermore, the majority of the species examined showed low levels of parthenogenetic development at least to an early embryonic stage. Thus, the capacity to at least initiate parthenogenetic development seems widespread in the genus. With such a small fraction of the total number of *Drosophila* species having been surveyed, it would not be surprising if more exhaustive sampling revealed additional cases like *D. mangabeirai*.

**Table 1:** Drosophilids in which parthenogenetic development has been screened or in which parthenogenetically reproducing strains were successfully created by laboratory selection (shaded)

Subgenus	Species Group	Species (Reference)	No. Eggs Examined	Embryos/Larvae Observed	No. Adults
Sophophora	Melanogaster	*D. melanogaster* (S54)	532,197		
		*D. melanogaster* (F86)			Selected
		*D. simulans* (S54)	13,872	1/0	
		*D. ananassae (*F72)			Selected
		*D. pallidosa (*F 9)			Selected
		*D. pallidosa-like* (M&T99)			Selected
	Obscura	*D. affinis* (S54)	19,059	4/0	1
	Willistoni	*D. willistoni (*W64)	NA		
		*D. paulistorum* (W64)	NA		x
		*D. equinoxlais* W64	NA		
		*D. tropicalis* (W64	NA		
		*D. insularis* W64)	NA		x
		*D. cubana* (W64)	NA		
		*D. mangabeirai (*C,W&H57)			Obligate
Drosophila	Cardini	*D. cardini* (S54)	52,850	3/0	
		*D. polymorpha* (S 54)	37,629	109/0	Selected
		*D. neocardini* (S54)	11,439	1/0	
		*D. cardinoides* (S54)	30,777	3/0	
		*D. acutilabella* (S54)	16,463	11/0	
		*D. campestris* (S54)	23,682	14/0	
		*D. parthenogenetica* (S53)			Selected
	Funebris	*D. funebris* (S54)	43,198	3/0	
		*D. macrospina* (S54)	2655	0/0	
	Melanica	*D. melanica* (S54)	5199	1/1	
		*D. nigromelanica* (S54)		0/0	
	Robusta	*D. robusta* (C61)	10,585,000		14
		*D. robusta* (S54)	10,706	2/0	
	Immigrans	*D. immigrans* (S54)	8153	1/0	
		*D. albomicans* (O&F95)			Selected
	Virilis	*D. americana* (S54)	18,165	0/0	
	Quinaria	*D. quinaria* (S54)	11,868	6/0	
		*D. transversa* (S54)	11,616	2/0	
	Testacea	*D. putrida* (S54)	8431	4/0	
	Repleta	*D. hydei* (T79)	381,715	3/3	2
		*D. hydei* (S54)	63,027	18/1	
		*D. hydei* (H62)	214,640	X/8	2
		*D. mercatorum* (C67)			Selected
		*D. mercatorum* (T79)	611,086	9/9	7
		*D. longicornis* (H62)	33,060	X/0	
		*D. aldrichi* (H62)	0	0/0	
		*D. hamatofila* (H62)	145,220	X/3	
		*D. hydeoides* (H62)	15,520	X/1	
		*D. meridiana* (H62)	37,580	X/0	
		*D. m. rioensis* (H62)	8620	X/1	
		*D. mulleri* (H62)	89,140	X/4	1
		*D. spenceri* (H62)	12,400	X/0	
Hirtodrosophila		*D. duncani* (S54)	16,044	1/0	
Scaptodrosophila		*D. victoria* (S54)	1292	0/0	
Scaptomyza		*graminum* (S54)	4314	4/0	
		*Adusta* (S54)	2340	0/0	
Zaprionus		*Z. vittiger* (S54)	10,167	0/0	

Number of individuals observed to undergo embryonic or larval development is given either as the number reported or an “x,” indicating that it had been observed but not reported as a number. C61, Carson 1961; CW&H 75, Carson *et al.* 1957; F72, Futch 1972; F79, Futch 1979; F86, Fuyama 1986a; H62, Henslee 1974; M&T99, Matsuda and Tobari 1999; NA, not available; O&F95, Ohsako and Fuyama 1995; T79, Templeton 1979; S53, Stalker 1952; S54, Stalker 1954; W64, Winge 1965.

*D. mangabeirai* represents the only truly parthenogenetic species observed in nature. This rare species is a member of the willistoni group. It has been collected in tropical regions of Brazil, Trinidad, and Central America, but of the total of 116 specimens collected, only three males were found, none of which were fertile and all of which probably were XO (Carson *et al.* 1957; Carson 1962). Although no cultures presently exist, two independent strains, each derived from a single female, were established and maintained for long periods in the laboratory (Carson 1962). Unlike parthenogenetic strains of other *Drosophila* species developed by laboratory selection, in which only approximately 1–2% of the eggs hatch, hatching of *D. mangabeirai* eggs is well over 60% and approximately 50% eggs survive to adulthood (Carson *et al.* 1957). Of 4362 F1 adult offspring emerging in the laboratory, none were male (Carson 1962). None of field-caught females had sperm in their seminal receptacles or spermathecae. Furthermore, of 270 randomly chosen larvae, salivary configurations all showed heterozygosity for three chromosomal inversions (Murdy and Carson 1959; Carson 1962), a large part of the genome.

## From facultative to complete parthenogenesis in *Drosophila*

If 1 in 40 species surveyed is a female-only species, why are there not more? The answer probably lies in the complexity of the underlying mechanisms and only a species in which the “perfect storm” is present will become parthenogenetic. What are the components of the “perfect storm?” Several seem obvious:

### Oviposition without insemination

Previous reviews of *Drosophila* parthenogenesis (Stalker 1954; Templeton 1983) focused upon the roles of meiosis and early postmeiotic development in successful impaternate development. But even earlier in the sequence of events is the requirement that females oviposit unfertilized eggs. In *Drosophila melanogaster*, meiosis is completed when the egg is laid (Doane 1960). Although virgin *D. melanogaster* females will lay small amounts of unfertilized eggs, male ejaculatory proteins passed during copulation are known to significantly increase oviposition (reviewed in Wolfner 2002). Oviposition by virgin females has not been systematically quantified in other *Drosophila* species, but unpublished evidence suggests considerable within- and between-species variation in the dumping of large numbers of unfertilized eggs. For example, virgin *Drosophila hydei* females can effectively coat the surface of a food vial with unfertilized oocytes. In other species, such as *Drosophila mojavensis sonora*, unmated females can harbor several stage 14 oocytes per ovariole but not lay them until after they mate (T. A. Markow, unpublished results). Thus, females of some species require a signal, probably a seminal fluid molecule or the mechanical stimulation of copulation to oviposit, whereas in others they do not. The connection between mating and oviposition thus remains a critical but unexplored component of successful parthenogenesis. Indeed, parthenogenesis was first discovered in the cardini group because Stalker (Stalker 1951, 1952) interspecifically mated females in an effort to study reproductive isolation. His interspecific crosses were infertile, but in some cases, mating to a heterospecific male was sufficient to trigger oviposition of unfertilized eggs that subsequently gave rise to parthenogenetically produced adult female flies. Given the low success of parthenogenetic development in all *Drosophila* species screened with the exception of *D. mangabeirai*, it follows that an initial prerequisite for the evolution of parthenogenesis is oviposition by virgin females.

### Formation of a diploid embryo

Terminology for cytological mechanisms leading to parthenogenesis was first discussed by White (1945) and Suomalainen (1950), and they fall into two categories: automixis and apomixis. Apomixis, more common in plants than in insects, typically involves only one maturation division, a division that is equational rather than reductional. Apomixis results in offspring that are genetically identical to the parents, whereas automixis produces genetically different offspring. Thus, the diploid chromosome number is retained. No evidence for apomictic mechanisms exists in *Drosophila*. Automictic mechanisms restore diploidy either through replication and subsequent fusion of the egg pronucleus or a polar body, the fusion of the egg pronucleus with a polar body nucleus, or the fusion of two polar body pronuclei. In *D. parthenogenetica*, cytological and genetic studies suggest that diploid eggs form from the fusion of two polar body nuclei and thus formation of the polar bodies, which can be variable, is critical for subsequent fusion (Stalker 1954). A detailed cytological study of development of unfertilized eggs from females in parthenogenetic laboratory strains of *Drosophila mercatorum* revealed a critical role of centrosome quality, quantity, and position in successful restoration of diploidy and early embryogenesis (Eisman and Kaufman 2007). Unlike *Nasonia*, in which centrosomes form *de novo*, *D. mercatorum* lack the paternal contribution to early spindle formation. This in turn derails the restoration of diploidy and/or early cleavage divisions, leading to the failure of the majority of eggs to develop. Although not examined cytologically, this is likely to be the case with *D. polymorpha* as well, because like *D. mercatorum*, after generations of selection for parthenogenesis, only a low percentage of eggs successfully developed (Stalker 1954). In parthenogenetic *Drosophila ananassae* and *Drosophila pallidosa*, diploidy is restored by postmeiotic nuclear doubling of a single meiotic product (Matsuda and Tobari 2004), different from the mechanism reported for *D. melanogaster*, where fusion between non-sister nuclei following second division restores diploidy (Fuyama 1986a).

Early events in *D. mangabeirai* eggs differ from the other *Drosophila* that show parthenogenesis in several ways, and these differences likely underlie its ability to produce adult female offspring from the majority of their eggs. The meiotic spindle in *D. mangabeirai* oocytes assumes a longitudinal rather than a transverse orientation as in the *D. melanogaster* spindle and tends to be located deeper in the ooplasm (Murdy and Carson 1959). The *D. mangabeirai* study was performed more than 50 years ago, however, and lacked the sorts of details about centrosomes provided for *D. mercatorum* (Eisman and Kaufman 2007). The high developmental success rate of *D. mangabeirai* eggs suggests that they have somehow solved the centrosome issues problematic for *D. mercatorum*.

### Embryonic survival

Aborted development in the species screened (Table 1) was observed primarily during the embryonic stage or in post-hatching first instar larvae. Postzygotic lethality in facultatively parthenogenetic species has been studied in detail only in selected strains of *D. mercatorum* (Eisman and Kaufman 2007), where approximately 4% of eggs yielded adult flies and 96% of the individuals died as embryos or early larvae. Of those few surviving the early larval period, approximately 20% subsequently die as late larvae. An earlier study (Sprackling 1960) suggested that early embryonic death in *D. parthenogenetica* is associated with ploidy deviations stemming from the particular pronuclei involved in the first cleavage division as well as their location within the egg. Deeper location was suggested to be associated with greater developmental success.

*D. mangabeirai* has overcome these early developmental problems: approximately 50% of eggs produce viable adults. The unique spindle orientation during meiosis appears to be important to successful fusion and early cleavage and for the reduced occurrence of the aberrant ploidy frequently observed in other *Drosophila* parthenogenetic embryos (Sprackling 1960; Eisman and Kaufman 2007). In addition, however, all viable *D. mangabeirai* larvae examined also were heterozygous for chromosomal inversions (Murdy and Carson 1959). If two meiotic product nuclei fused at random, and half of these were inversion heterozygotes, it could explain the percentage of eggs surviving to adulthood. Any means of creating balanced heterozygosity, eliminating mortality from recessive lethals, would increase successful development of *D. mangebeirai* embryos compared with the other species, in which homozygosity increases with each generation (Carson *et al.* 1969).

Early embryonic development in *Drosophila* is controlled maternally until the zygote genome is activated. Maternally contributing factors in the egg-to-embryo transition in fertilized oocytes thus also may be important in early development of unfertilized eggs. Several of these factors have been characterized, such as the YA (Sackton *et al.* 2009) and WISPY (Cui *et al.* 2008) proteins in *D. melanogaster*. The genes *sra* (Takeo *et al.* 2006), *cortex* (Swan and Schupbach 2005; Vardy *et al.* 2009) and the gn/plu/png complex (Lee *et al.* 2003) are involved in egg activation, the completion of meiosis, and early embryonic divisions. Because so many of the unfertilized eggs that undergo some development abort as very early embryos, variation loci that control very early developmental transitions, such as those discussed by Tadros and Lipschitz (2009) seem likely candidates for promoting or halting the parthenogenetic process.

## Evidence of a genetic basis for *Drosophila* parthenogenesis

Multiple lines of evidence suggest a genetic basis to the ability to undergo parthenogenetic development in *Drosophila*: the phylogenetic distribution of species exhibiting the trait, within-species differences in the frequency of parthenogenetic development, selection experiments, and mapping studies.

**Figure 1:**
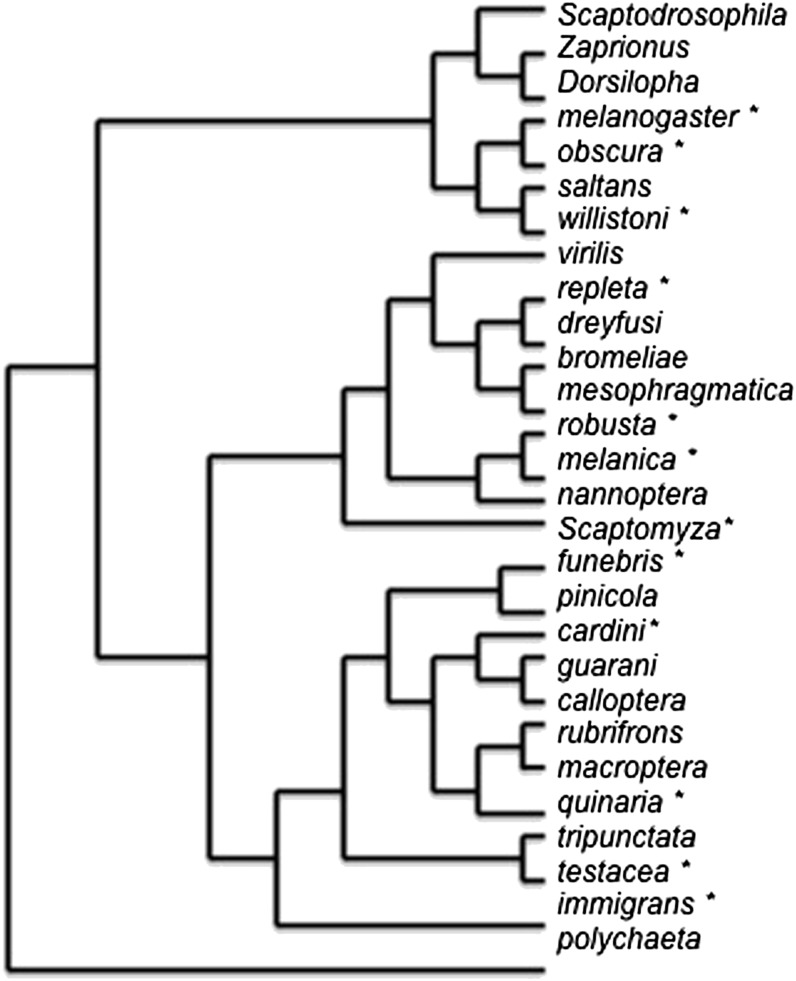
The phylogenetic relationships of the species groups in the genus *Drosophila* and related species in which at least some parthenogenetic development has been observed (*).

Screening for parthenogenesis has yet to be performed for all lineages and groups in the genus (Figure 1, Table 1). Both major subgenera, the Sophophora and Drosophila, however, contain species capable of at least initiating impaternate development (Figure 1). Among the Sophophora, *D. ananassae* and its sister species *D. pallidosa* are members of the melanogaster species group. Although a parthenogenetic strain of *D. melanogaster* exists, it was created artificially and the occurrence of natural parthenogenetic development is extremely low in this species and in *Drosophila simulans*. In the willistoni species group, rare parthenogenesis was observed in *Drosophila paulistorum* and *D. insularis* whereas their relative, *D. mangabeirai*, has become a female-only species. *Drosophila affinis*, of the obscura group, also showed parthenogenesis, but other obscura group species have not been screened. The subgenus Drosophila also contains many species in which some parthenogenetic development has been observed. Stalker (1951, 1952) had focused heavily on the cardini species group and Henslee (1974) on the repleta group, to which the selected strains of *D. mercatorum* belong. For other species group in the Drosophila subgenus, surveys have been limited, but in several cases, *D. albomicans* (Ohsako and Fuyama 1995), *D. robusta* (Carson 1961), *D. mercatorum* (Carson 1967), *D. parthenogenetica*, and *D. polymorpha* (Stalker 1954) selection effectively used existing variation in creating parthenogenetic laboratory strains. The majority of these species were never examined cytologically, so characteristics of spindle orientation, composition, and pronucleus location are unknown and cannot be compared with *D. mangabeirai* or *D. mercatorum*, where they have been well-described.

The variation among extant species must have arisen from ancestral genetic variation at loci controlling a range of relevant processes. Significant population-level variation in the frequencies at which unfertilized eggs will begin development has been reported in *D. mercatorum* (Templeton *et al.* 1976), *D*. *parthenogenetica* (Stalker 1954), *D. robusta* (Carson 1961), and *D. ananassae* (Futch 1972, 1973) reflects intraspecific variation in genetic propensities to undergo parthenogenesis.

Attempts in some species, such as *D. hydei*, to select for parthenogenesis have not been successful (Templeton 1979). However, laboratory selection has been successful in eight species and in one genetically constructed strain of *D. melanogaster*. Despite large increases in the number of eggs undergoing some development, decades of selection have never increased the proportion of eggs reaching adulthood beyond 8%. These gains largely reflect increases in the number of eggs undergoing any degree of development rather than gains in survival at later stages. In *D. parthenogenetica*, for example, 17 generations of selection produced a 20-fold increase in survival of eggs to the early larval stage, but the biggest increase was in the initiation of development, which still typically aborted in the embryonic state (Stalker 1954). The smaller increase in postmeiotic development may be attributable to variability in the mechanisms that restore diploidy, some of which, as discussed previously, favored more normal early development. Developmental failures past the early larval stage in selected strains probably reflect underlying deviations in ploidy or homozygosity for early-acting recessive lethals.

The observation that the *D. mangebeirai* meiotic spindle assumes an unusual orientation in the majority of embryos (Murdy and Carson 1959) suggests that selection favored this spindle orientation in overcoming early barriers to parthenogenetic embryogenesis. If facultatively parthenogenetic species lack sufficient genetic variation for processes underlying centrosome formation, behavior, and spindle orientation, it may explain why, even after decades of laboratory selection, only a small proportion of impaternate eggs produce adult females in most species.

Whether allelic variation is maintained at the relevant loci by mutation alone or by some selective advantage for asexual reproduction is not known. However, in populations of some species, such as *D. mercatorum*, as many as 27% of wild caught females were reported to be able to reproduce parthenogenetically (Templeton *et al.* 1976), suggesting that the responsible alleles are maintained in fairly high frequency, at least in some populations of this species.

The loci at which this allelic variation occurs is unknown. One can easily envision, however, that loci affecting oviposition of unfertilized eggs, completion of meiosis, restoration of diploidy, egg-to-embryo transitions, all could be important. Recent mapping studies have pointed to particular chromosomal locations of factors favoring parthenogenesis. Matsuda and Tobari (2004) reported a region on the *D. ananassae* second chromosome that contributes significantly to parthenogenesis and Fuyama (1986b) reported regions on the *D. melanogaster* second and third chromosomes contributing to parthenogenesis in an artificially constructed strain. In the case of *D. melanogaster*, the implicated regions were approximately 20 cM long, precluding any conclusions about the importance of a particular site as well as speculation as to any homology between the two species. *D. melanogaster* also can produce parthenogenetic adults tracing back to a defect in the first meiotic division (Meyer *et al.* 2010).

## Population level and ecological factors

Another factor that could mitigate or allow parthenogenetic development to proceed, once a diploid zygote has been created, is the genetic structure of the population of the species in which the event has occurred. Because parthenogenesis reduces genetic variability, the presence of deleterious recessives segregating in a population could result in the death of impaternate individuals at embryonic or later stages. Species and even different populations of the same species may differ in their relevant genetic backgrounds and genetic loads depending upon their own evolutionary histories. The fact that in *D. mangabeirai* all individuals are heterozygous for chromosomal inversions supports the role of homozygosity in the death of so many parthenogenetic embryos and early larvae in selected strains of *D. mercatorum*, *D. parhtenogenetica*, *D. polymorpha*, and *D. ananassae*.

Mating system features also could play a significant role in the appearance of parthenogenesis in certain species. In some *Drosophila* species, severe sperm limitation for females is turning out to be common in the laboratory and in the wild (Markow *et al.* 2012). This is especially true of species in which males produce giant sperm and pass very few gametes to females. Males of these species also require many days to become sexually mature, sometimes up to 2 or 3 wk, as in *D. pachea* and *D. bifurca*, respectively. These same species also are characterized by frequent female remating, which greatly boosts their sperm supply and offspring production. Females of sperm-limited species don’t have fewer ovarioles or make fewer eggs. The ratio of receptive females to sexually mature fertile males thus is very different in highly sperm-limited species compared with *D. melanogaster:* there are not enough sperm or males to go around. Added to this is the fact that population sizes differ among *Drosophila* species. In species with smaller population sizes, the probability of encountering a mate (Gowaty and Hubbell 2009) may be low, especially at certain times of the year or in certain parts of the species range. The ability to reproduce even a small number of impaternate female offspring, even for one generation, may mean the difference between extinction and survival of a population or species. It is easily imaginable that parthenogenesis could be favored in species in which females are frequently faced with mate or sperm shortages.

The foregoing are issues that can confront members of any taxon, but most other taxa are not as easily studied as in *Drosophila*. Furthermore, because of the growing information about the highly diverse natural history of different *Drosophila* species, and the development of genetic resources for these species, we are now better able to approximate the ecological and evolutionary features of many nonmodel taxa with flies. Specific steps and processes can be identified at which parthenogenesis may proceed or abort: (1) females have to oviposit without insemination, (2) diploid zygotes must be produced, (3) early cleavage and blastoderm formation must be normal, and (4) the remainder of embryogenesis and postembryonic development must be normal. At this point not only is normal cell division required, but also a means of avoiding death from later-acting recessive lethals. At least one *Drosophila* species, *D. mangabeirai*, has solved these problems (Carson *et al.* 1957). Understanding how they accomplish this will require recollecting the species from the wild. Screening additional species may reveal more like *D. mangabeirai*. Genomic approaches, including comparing genomes of parthenogenetic and sexual strains of the same species and examining expression patterns during the early developmental stages, should help reveal the mechanisms underlying the failure of the impaternate embryos in some *Drosophila* species to complete development.
